# Supplementary material for: A Soft Zwitterionic Hydrogel as Potential Coating on a Polyimide Surface to Reduce Foreign Body Reaction to Intraneural Electrodes
Source: Molecules. 2022 May 13;27(10):3126. doi: 10.3390/molecules27103126 (PMC9147366; doi:10.3390/molecules27103126)
Supplement: Supplementary file 1 [file molecules-27-03126-s001.zip › molecules-1690909-supplementary.pdf]

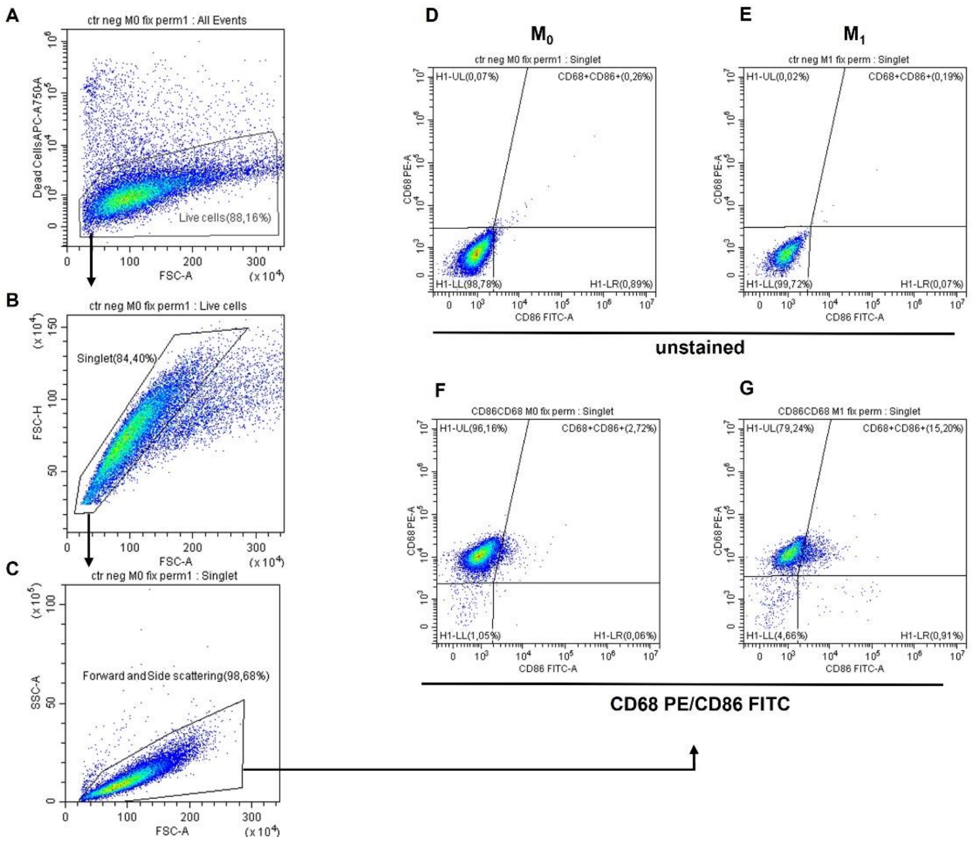

Figure S1. $CD68^+CD86^{-/low}$ M_0-macrophage and $CD68^+CD86^+$ M_1-macrophage phenotypes. Representative flow cytometry gating of live cells for the M_0 macrophages (negative ctrl) in (**A**) as well as forward (FSC) and side scatter (SSC) intensity plots to gating Singlet in (**B**) and morphological analysis in (**C**). Evaluation of M_0 (left) and M_1 (right) cell phenotypes (**D**–**G**) was determined by flow cytometry as percentage for the expression of the pan-macrophage marker CD68 and the specific M_1 marker CD86. We reported the two-color fluorescence dot plots of a representative experiment. The percentage of double positive cells in M_1 (H1-UR quadrant in (**G**)) shows a 5.6-fold increase compared to M_0 cells (H1-UR quadrant in (**F**)).
